# Selenite elimination *via* zero-valent iron modified biochar synthesized from tobacco straw and copper slag: Mechanisms and agro-industrial practicality

**DOI:** 10.3389/fbioe.2022.1054801

**Published:** 2022-11-14

**Authors:** Qiong Luo, Dingxiang Chen, Ting Cui, Ran Duan, Yi Wen, Fang Deng, Lifang Li, Huabin Wang, Yong Zhang, Rui Xu

**Affiliations:** ^1^ School of Energy and Environment Science, Yunnan Normal University, Kunming, China; ^2^ Yunnan Key Laboratory of Rural Energy Engineering, Kunming, China

**Keywords:** selenite removal, modified biochar, copper slag, tobacco straw, practical feasibility

## Abstract

Cost-effectively improving the performance of biochar is essential for its large-scale practical application. In this work, the agro-industrial by-products copper slag and tobacco straw were employed for the preparation of modified biochar (CSBC). The obtained CSBC exhibited satisfactory capacity on Se(IV) immobilization of 190.53 mg/g, with surface interactions determined by the monolayer and mainly chemisorption. The removal mechanisms included chemical reduction, electrostatic attraction, co-precipitation, and formation of complexations. Interestingly, the existence of Cu_2_Se structure after adsorption indicated the involvement of Cu species within Se(IV) elimination. Moreover, the industrial agricultural practicality of CSBC was evaluated by regeneration tests, economic assessment, and pot experiments. The results demonstrate that iron species-modified biochar prepared from two agro-industrial by-products is a promising and feasible candidate for selenite removal from wastewater.

## Introduction

Selenium (Se) is an indispensable element for human beings, but excessive Se intake could be detrimental, resulting in liver damage, reproductive failure, and hair and nail loss (Kushwaha et al., 2022). The main chemical states of Se that exist in wastewater are selenide, elemental selenium, selenate, and selenite, which are referred as Se^2-^, Se^0^, SeO_3_^2-^, and SeO_4_^2-^, respectively (Li J et al., 2021). Among these species, Se(IV), which normally exists as SeO_3_^2-^, is the most toxic for human beings because of its lower mobility and negligible bio-degradability (Xiong et al., 2022).

The main sources of selenium pollution in the environment include both natural and anthropogenic sources. The natural sources of Se mainly include volcanic eruptions and biogeochemical processes of selenium-containing rocks. Volcanoes can shoot large amounts of toxic metal elements (including Se) into the environment (Li, et al., 2022). The main anthropogenic activities leading to Se pollution are mining, fossil fuel burning, fertilization, and precious metal processing. The concentration of Se(IV) in wastewater ranges from 0.2 to 74 mg/L (Zhang X et al., 2022).

Biochar derived from biomass is widely employed as an environmental functional material due to its high functionality, cost-effectiveness, and environmentally benign nature (Zhu et al., 2021; Yang et al., 2022). However, the major bottleneck of biochar-based adsorbents is their limited removal capacity. Consequently, many researchers have attempted to develop an economically feasible method to modify biochar to enhance its adsorption performance and introduce a pilot-scale application for Se(IV) pollution control (Liang et al., 2021).

Iron (Fe) is considered one of the most available and abundant elements on the Earth and many different iron-based materials have been employed for biochar fabrication (Xu et al., 2022). On one hand, the existence of Fe in biochar could enhance its removal capacity due to surface interactions between pollutants and these chemically reactive species. On the other hand, Fe-contained materials could offer more functionalities, such as magnetism and chemically reductive property (Yang X et al., 2021). Magnetic nanomaterials are superior adsorbents in water remediation application (Abdel Maksoud et al., 2020). Our group applied FeCl_3_ to prepare magnetic biochar for Hg(II) removal, and the capacity reached 167.22 mg/g (Wang L et al., 2018). Other metal slats are also commonly employed for biochar modification, such as Fe(NO_3_)_3_, MgFe_2_O_4_, K_2_FeO_4_, and FeSO_4_ (Wang Q et al., 2021; Yang F et al., 2021; Wen et al., 2022). However, their high price violates the principle of cost-effectiveness and hinders the practical application of these biochar materials. To solve this problem, there is now a novel trend toward applying iron-containing solid wastes as an iron source for biochar fabrication, such as municipal sewage sludge (Ifthikar et al., 2017), red mud (Wang J et al., 2020 (Wang, et al., 2022a)), aluminum residues (Zhao R et al., 2021), steel pickling waste (Yi et al., 2021), and steel slag (Wang, et al., 2022b). However, the influence of major impurities in these Fe-contained by-products on biochar properties has mostly been ignored (e.g., aluminum in red mud and phosphate in sludge), which urgently needs to be analyzed (Godlewska et al., 2021).

The copper metallurgy industry generates a large amount of copper slag (CS); usually 2.2–3.0 tons of CS is produced for 1 ton of Cu production (Zhao Z et al., 2021). The iron content in CS is generally close to 40 wt%, while the remaining part is Si, Cu, Al, Ca, and so on (Mikula et al., 2021). Currently, CS is normally applied for building construction, such as road paving, or as a substitute for cement. Meanwhile, efficient reutilization of Fe species in CS has attracted little attention (Li Y et al., 2021). Recently, researchers have attempted to reutilize CS as replacement for conventional iron salts to modify biochar. Gao et al. (2021) applied CS to prepare porous silicate supported Fe^0^ to activate persulfate and remove orange G. They employed 20% anthracite as a reductant during co-pyrolysis at a temperature of 1100°C, which provided a novel approach for the synthesis of Fe activators *via* this carbothermal reduction process. Hence, CS could be employed for biochar fabrication, which could reduce the preparation cost for the synthesis of efficient and effective bio-adsorbents and further benefit their large-scale application. Tobacco straw is a bio-resource that is generated during cigarette production, whose reutilization and recycling has been less reported (Wang et al., 2019). This straw could be applied as a precursor for biochar preparation. Its high cellulose content and the lignin in its structure gives it a large surface area and a significant increase of microporous and mesoporous structures, which can be used as an effective place to capture target pollutants (Osman, et al., 2022b). This might be of benefit to Se(IV) removal. Therefore, a promising solution could be to use CS as an iron source and carbothermal reduced by tobacco straw to prepare functional biochar for Se(IV) pollution control.

In this work, copper slag and tobacco straw were employed as raw materials for preparation of Fe^0^-modified biochar (CSBC) and then applied for Se(IV) removal from wastewater. The physicochemical properties of the obtained CSBC were comprehensively investigated to evaluate the loading of chemically reactive species. The Se(IV) removal capacity was then tested, and thermodynamics analyses were employed to illustrate the surface interactions. Several techniques have been adopted to elucidate the reaction mechanisms. Finally, recycling test, economic assessment, and pot experiments were conducted to evaluate the field feasibility of this environmentally functional material.

## Materials and methods

### Materials and regents

The copper slag (CS) that was applied in this study was collected from Xuanwei Smelting Plant, Yunnan Province (104.10°E, 26.22°N). The CS powders were washed with DI water three times and then dried in an oven overnight at 60°C. They were then crushed manually and passed through 200 meshes for further analysis. The chemical elements of CS are listed in Table 1. We found that there were 5.84% Cu, 18.32% Si, 40.52% Fe, and 0.89% Ca in the CS. The tobacco straw that was applied in this study was collected from rural areas near the Yunnan Normal University. They were washed with DI water and stored for 12 h at 60°C. After it was dried, the biomass was smashed and sieved *via* 200 meshes. The chemical reagents—including ethanol, NaOH, HNO_3_, HCl, and Na_2_SeO_3_—were all obtained from Sinopharm Co. Ltd. and used without further purification.

**TABLE 1:** Chemical constitution of CS (X-ray fluorescence data).

Element	Fe	Cu	Si	Al	Ca	Mg	Na	K	O	As	Zn
Constitutes (wt%)	40.52	5.84	18.32	4.35	0.89	0.74	1.35	1.58	30.14	0.24	2.58

### Preparation of CSBC

**SCHEME 1:**
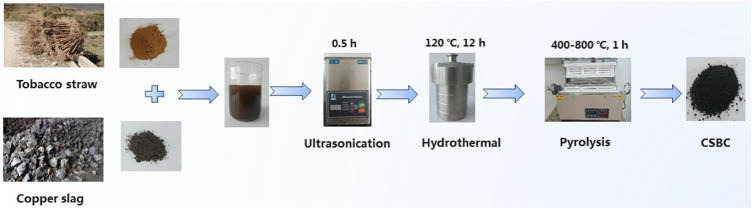
Scheme of the preparation process of CSBC.

The fabrication process that was used for CSBC is given in Scheme 1. Carbothermal reduction was employed for preparation of CSBC, which was modified from our former work (Wang H et al., 2020). Briefly, 4.0 g CS and 2.0, 4.0, and 8.0 g tobacco straw were added to the 40 ml NaOH solution (0.5 M). The mixture was ultra-sonicated for 0.5 h and then stirred magnetically for another 2 h. The slurry was then transferred into an autoclave for hydrothermal treatment for 10 h at 140°C. The high temperature and autogenous high pressure under hydrothermal action ensures a good consistency between additives and bases. After cooling, the slurry was poured out, filtered, and the solid parts were dried. The solid part was then placed in a furnace for pyrolysis, the temperature was set at 400–800°C and kept for 1.0 h, the increasing rate was 5°C/min, and the whole process was protected with nitrogen (99.999%). The obtained biochar was washed with ethanol and DI water and dried in a vacuum oven for further tests. The CSBC prepared in this study is labeled as CSBCX-Y, where X refers to the pyrolysis temperature and Y represents the weight ratios between CS and biomass, such as CSBC800-0.5. The weight ratio between CS and tobacco straw was 0.5: 1, while pyrolysis was under 800°C.

### Characterization methods

The surface morphology of CSBC was observed by scanning electron microscopy equipped with energy-dispersive spectroscopy (SEM-EDS, Hitachi Regulus8100, Japan). The micro-structure and surface area of the adsorbents were investigated by nitrogen adsorption–desorption isotherms (ASAP2460, Micrometrics, United States) and calculated by the Brunauer–Emmett–Teller (BET) method. Fourier transform infrared spectroscopy (FT-IR, Thermo scientific Nicolet iS 10, United States) was employed to analyze the surface functional group on CSBC with the conventional KBr method. The crystalline structure was investigated by X-ray diffraction (XRD, Nalytical X'Pert PRO MPD, Holland), with the radiation of Cu–Kα and a scanning rate of 5°/min. X-ray photoelectron spectroscopy (XPS, Thermo Scientific ESCALAB 250XI, United States) was used to analyze the chemical states of the surface elements of the CSBC. The chemical composition and elemental concentration of the CSBC were tested with an atomic adsorption spectrophotometer (AAS, Shimadzu AA7700, Japan) and X-ray fluorescence spectrometer (XRF, PANalytical Axios, Holland), respectively. The concentration of heavy metals was digested with 1 M HNO_3_, diluted, and tested by using an Inductively Coupled Plasma Optical Emission Spectrometer (ICP-OES, PerkinElmer 8300, United States). Each sample was analyzed in triplicate to ensure the integrity of the data.

### Batch experiments

CSBC samples were placed into serum bottles with a dosage of 2.0 g/L, and 10 ml Se(IV) solution was applied for capacity evaluation at an initial concentration of 10 ppm. The mixture was sealed and stirred for 24 h at a speed of 200 rpm and at room temperature (25°C). The effects of initial solution pH (2.0-9.0) and adsorbent dosage (10–40 g/L) were also investigated with similar procedures. The concentration of residual Se(IV) species was tested by ICP-OES, and a 0.22-μm filter was used to collect the supernatant before testing. Se(IV) removal capacity (Qe, mg/g) and efficiency ( η ) by CSBC were calculated by the following equations:

$$\eta =\frac{\left(C_{0}-C_{t}\right)}{C_{0}}*100\%,$$ (1)

$$Q_{e}=\frac{\left(C_{0}-C_{t}\right)V}{m},$$ (2)

where C_t_ (mg/L) and C_0_ (mg/L) represent the Se(IV) concentration at time t and 0, respectively; V (ml) and m (mg) represent solution volume and mass of adsorbents, respectively.

Kinetics analysis was adopted with an initial solution concentration of 50 ppm, a dosage of 2.0 g/L, and the reaction was conducted at 25°C and tested with different time intervals (from 0.1 h to 14 h). The pseudo-first-order (Eq. 3) and pseudo-second-order (Eq. 4) models were employed for data fitting:

$$Q_{t}=Q_{e}\left(1-e^{tk_{1}}\right),$$ (3)

$$Q_{t}=\frac{k_{2}Q_{e}^{2}t}{1+k_{2}Q_{e}t,}$$ (4)

where t (min) represents reaction time, Q_t_ (mg/g) represents the selenite uptake at time t, and k_1_ and k_2_ represent the rate constant for the pseudo-first-order and pseudo-second-order model, respectively.

The adsorption isotherms were conducted as the Se(IV) initial concentration varied from 10 to 400 mg/L, the initial pH was 2, adsorbent dosage was 2.0 g/L, and was reacted for 24 h at room temperature (25°C). The Langmuir (Eq. 5) and Freundlich (Eq. 6) models were applied to analyze the experimental data,

$$Q_{e}=\frac{K_{L}Q_{\max}C_{e}}{1+K_{L}C_{e}},$$ (5)

$$Q_{e}=K_{F}C_{e}^{1/n_{F},}$$ (6)

where K_L_ and K_F_ represent the isotherm constant for Langmuir and Freundlich models, respectively; n_F_ represents the parameter for adsorption intensity evaluation; and Q_max_ (mg/g) represents the maximum capacity calculated by models.

Thermodynamic parameters of enthalpy change (∆H), entropy change (∆S), and Gibbs free energy (∆G) can be calculated by the following formulas:

$$\ln\left(\frac{q_{e}}{c_{e}}\right)=\frac{∆S}{R}-\frac{∆H}{RT},$$ (7)

∆G=∆H−∆ST. (8)

### Recycling test and pot experiments

The regenerative capability of CSBC was confirmed by bath solution experiments. The common pyrolysis recycling method was applied for CSBC regeneration. The Se(IV)-loaded biochar was washed and then dried in a vacuum oven. The sample was then heated at 600°C for 1 h with the protection of nitrogen, and the obtained CSBC was directly applied to another cycle in the solution. The pyrolysis-based recycling method could regenerate chemically reactive species and also provide more adhering sites (Wang et al., 2022).

A series of soil experiments was employed to evaluate the practicality of the prepared CSBC on Se(IV) polluted soil remediation. The design of these experiments was based on the work of Man et al. (2021). In detail, 4 kg garden soil was added into each pot, and Se(IV) concentration was set as 100 mg/kg. Selenium slats were dissolved, and 50 ml solution was mixed into the uncontaminated soil to form different samples. These polluted soil samples were incubated for 30 days under room conditions to ensure the uniform distribution of the pollutants. Subsequently, different adsorbent materials—that is, CS, BC, CS + BC, and CSBC (1 g/kg)—were added into each pot to react with the contaminated species. After another 30 days of incubation, bok choy seeds (provided by Weifang city, Shandong Province) were planted in the experimental pots in duplicate. The growth status was recorded after 30 days of cultivation and the heavy metal content in the plants was tested by harvesting 1.0 g stems, leaves, and roots digested with concentrated HNO_3_, which were diluted and analyzed by ICP-OES.

## Results and discussion

### Physicochemical characterizations

The elementary distribution and morphology of prepared CSBC was observed by SEM-EDS (see Supplementary Data). The porous structure of BC was obtained indicating the feasibility of this biochar as a backbone for loading of CS functional species. After adding CS, obvious aggregated Fe ions loaded on to the biochar surface and the rough structure was maintained, and the porous surface and existence of Fe species provided sufficient reactive sites for contaminant adherence (Yan et al., 2021). The distribution of metals on the adsorbents was shown in EDS mapping images. The almost uniform distribution of Fe, Cu, Si, and Al indicates the successful preparation of CSBC. Moreover, the content of Fe (38.53%) was much higher than that of other metals, which is in agreement with the composition analysis results of CS.

The N_2_ adsorption–desorption isotherm was applied to evaluate the surface parameters of the obtained CSBC (see Supplementary Data). The hysteresis loop associated with the type III curve is based on the classification, while the desorption and adsorption data did not fully coincide. This demonstrates the abundant mesoporous structure of CSBC (Bao et al., 2021). Meanwhile, according to the BET calculation, the specific surface area of the obtained CSBC was 124.26 m^2^/g. The mesoporous dominated interior and rough surface structure of CSBC are beneficial to pollutant capture. It may also be beneficial to the reduction of pollutants (Fawzy, et al., 2022).

XRD was performed to analyze the crystal structure of CSBC (see Supplementary Data). The pattern reveals that the mineral composition of CSBC was complicated, which was in accordance with the chemical composition analysis. Fe_2_O_3_ with peaks at 31.28°, 33.01°, and 39.07° (PDF#01-073–0603) and Fe_3_O_4_ with peaks at 36.16, 52.34°, and 58.37° (PDF#01-089–0951) were the major Fe phases. Meanwhile, the peaks of Fe^0^ were detected at the 2θ of 44.69°and 64.23° (PDF#00-006–0696). This indicates that ZVI was successfully generated. The XRD pattern shows that the intensity of Fe^0^ peak increased with increasing temperature, which was related to more reducing gas being produced at high temperature and was conducive to the reduction of iron oxide to ZVI. In addition, the XRD image of the CS content (see Supplementary Data) shows that the Fe^0^ peak intensity of more CS had no obvious change. This may happen because the reducing gas generated in the biochar pyrolysis process can only reduce parts of iron oxide to ZVI.

The information of surface functional groups on the surface of adsorbents was analyzed by FT-IR (see Supplementary Data). The abundant peaks with various wavenumbers in CSBC indicate the rich components from the tobacco straw, such as 3345 cm^−1^ for the vibration of -OH groups, while the wavenumbers of 1632, 1396, 1080, and 872 cm^−1^ correspond to the existence of C=O, C=C, C-C, and C-H, respectively (Wang F et al., 2021). The vibration of Fe-O was observed in the case of 550 cm^−1^, demonstrating the loading of Fe species in the biochar substrate. All these results proved the abundant functional groups on the surface of adsorbents, which could react with the contaminants.

XPS can interpret the elemental chemical compositions on the adsorbent’s surface (see Supplementary Data). The characteristic peaks of C1s with the binding energy of 284.1, 285.4, 287.8, and 288.5 eV were associated with the structure of C=C, C-O, C=O, and O-C=O, respectively (Huang et al., 2021). These carbon structures were assigned with the FT-IR results. Furthermore, the peaks of Fe^0^ were observed with the binding energy of 719.3 eV. This indicates that a carbothermal reduction occurred and that CS successfully acted as an iron source. Moreover, the peaks of Cu^0^ in Cu 2p at 932.2 and 952.9 eV provided evidence of copper reduction from Cu(II) to Cu^0^, which might interact with selenite contaminants (Zhou et al., 2021).

### Influence of preparation parameters

**FIGURE 1:**
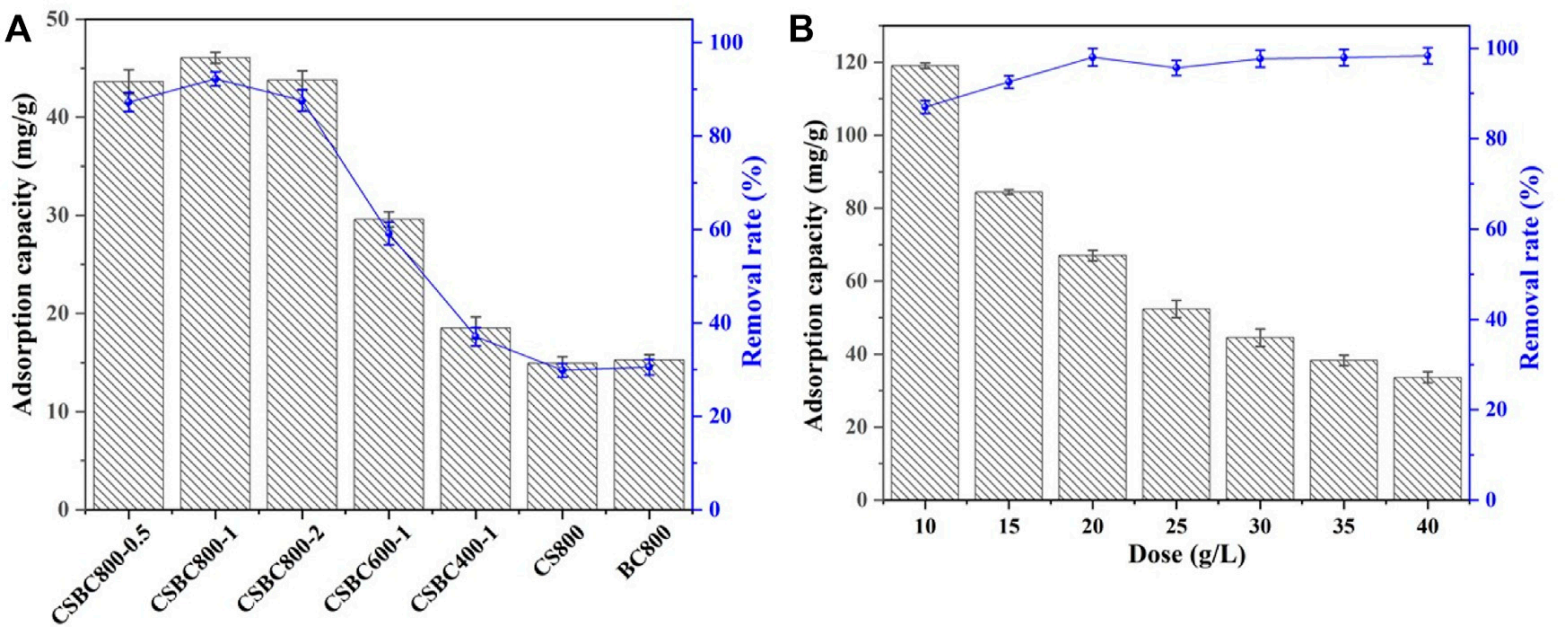
Adsorption capacity and removal rate of CSBC at different pyrolysis temperatures, weight ratios between CS and biomass **(A),** and different dosage **(B)**.

The effects of synthesis conditions of CSBC were analyzed, including pyrolysis temperature and weight ratios between components. As shown in Figure 1, the CSBC prepared with different temperatures exhibited obvious changes. As the pyrolysis temperature increased, the removal capacity increased from 23.53 mg/g of CSBC400-1 to 46.07 mg/g of CSBC800-1. This could be caused by the varying surface area and crystalline structure with CSBC synthesized at different temperatures. Moreover, the Se(IV) removal efficiency reached 92.20% for CSBC800-1, which indicates that this CSBC functional material is a potential and promising adsorbent for selenite pollution control.

The influence of weight ratios between CS and biomass was also investigated, as shown in Figure 1. According to the results, CSBC800-0.5 exhibited lower capacity (42.63 mg/g) than CSBC800-1 and CSBC800-2 (46.07 and 43.75 mg/g, respectively), but was higher than that of the pristine CS or BC. This proves the vital role played by the functional species provided by CS and also the synergistic effects between CS and BC after successful preparation. Interestingly, the capacity for CSBC800-1 and CSBC800-2 was 46.07 and 43.79 mg/g, respectively. This negligible decrease (4.88%) indicates the limitation for CS loading of the BC substrate—too much iron species could block the pore-structure of BC. This provides evidence for the multiple mechanisms of Se(IV) removal by CSBC.

### Se(IV) adsorption performance

Dosage is an essential factor for the adsorption processes. Based on the results (Figure 1), the removal efficiency increased from 87.01% to 98.35% as the adsorbent dosage increased from 10 to 40 g/L, respectively. This may have happened because there was more CSBC in the solution that provided the sufficient reactive sites for Se(IV) adherence, which contributes to the high removal efficiency of the system. However, when the CSBC dosage kept increasing from 10 to 40 g/L, the capacity dropped obviously from 119.04 to 33.67 mg/g, respectively. This is in accordance with our former reports, which showed that more CSBC leads to the capacity for per unit functional materials. Hence, based on this analysis, the CSBC800-1 was applied for further experiments. The adsorbent dosage was set as 2.0 g/L to get a balance between capacity and Se(IV) removal efficiency.

**FIGURE 2:**
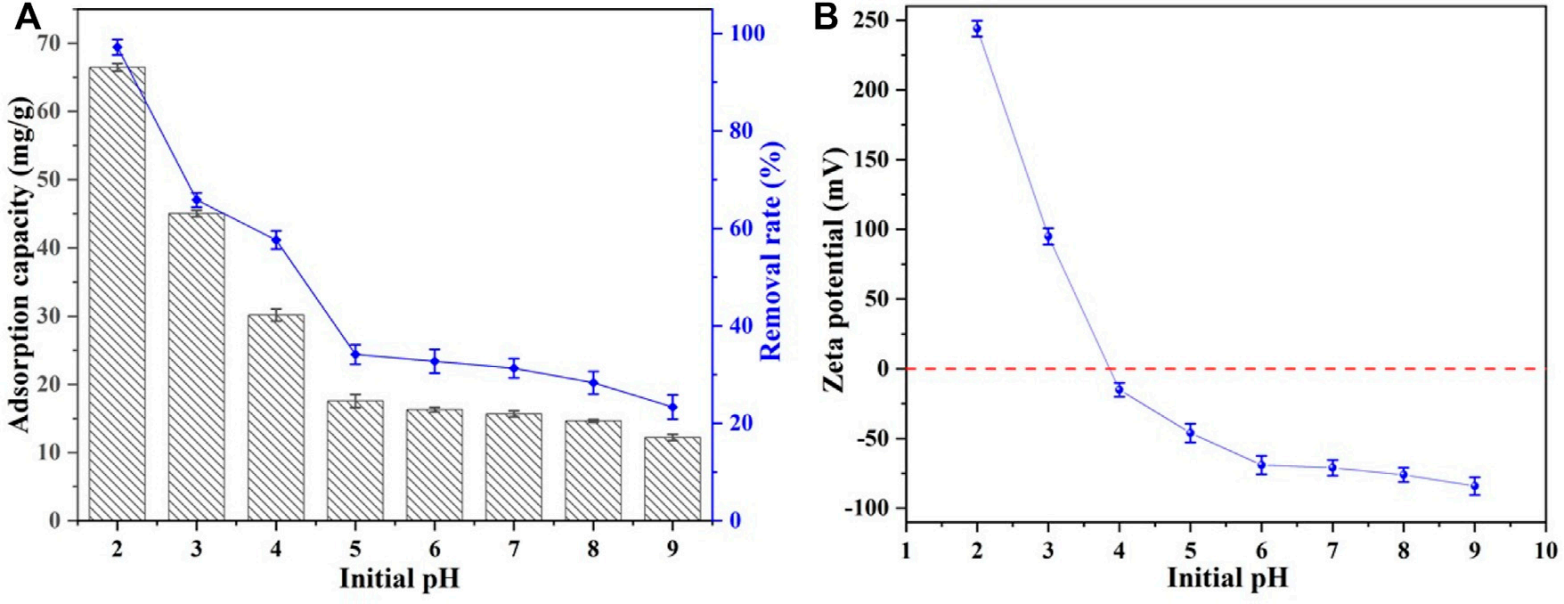
Adsorption capacity and removal rate of CSBC with different initial pH solution **(A)** and zeta potential **(B)**.

The influence of initial solution pH was considered an important parameter for surface interactions, as depicted in Figure 2. The removal capacity of Se(IV) by CSBC was decreased from 66.47 mg/g at pH 2 to 12.23 mg/g at pH of 9. This could be attributed to the better electron shuttling effects of Fe^0^ species under acid conditions (Liu et al., 2022). Moreover, the surface zeta potential of CSBC was tested (see Supplementary Data), and the pH_ZPC_ of CSBC was 3.91. When the solution’s pH moved beyond pH_zpc_, the surface was charged, and the anionic Se(IV) and sorbent surface repelled one another. This reduces the efficiency of Se(IV) elimination. When it decreased below pH_zpc_, the positive charge became a negative charge, which admirably adsorbs the cationic Se(IV) molecules (Osman, et al., 2022a). This indicates the positively charged adsorbent surface under acid conditions, which further benefits the attraction of SeO_3_^2-^ oxyanions. The maximum capacity of Se(IV) contaminants was 66.47 mg/g with pH 2. However, this severe acid situation barely exists in conventional water surroundings—Se-containing wastewater is generally acidic to weakly alkaline (3.0–8.0) (Zhang L et al., 2022). Hence, the initial solution pH in the following sections was set as 4. Therefore, electrostatic attraction could be one of the removal mechanisms, while more inter-surface reactions could be elucidated by thermodynamics analysis.

According to the kinetics study, Se(IV) removal by CSBC800-1 reached equilibrium within 240 min, with a capacity of 24.16 mg/g. The conventional models of pseudo-first-order and pseudo-second-order were applied to mimic the removal processes, and the results are listed in Table 2. The fitting parameters of the pseudo-second-order models exhibited better results, with a capacity of 24.54 mg/g and coefficient values of 0.996, when compared with the pseudo-first-order model (23.16 mg/g and 0.942, respectively), This is consistent with the previous research results that the adsorption of Se(IV) and Se(VI) by various adsorbents followed the quasi-second-order kinetic model (Tong, et al., 2022), indicating the interactions were dominated by chemisorption.

**TABLE 2:** Kinetic and isotherm model fitting parameters.

		Parameter 1	Parameter 2	*R* ^2^
Adsorption kinetics				
Pseudo-first-order		Q_m_ = 23.16 mg·g^−1^	K_1_ = 8.68 min^−1^	0.942
Pseudo-second-order		Q_m_ = 24.54 mg·g^−1^	K_2_ = 0.52 mg/g·min^−1^	0.995
Adsorption isotherm				
Langmuir	298K	Q_m_ = 353.62 mg·g^−1^	K_L_ = 0.0014 L·mg^−1^	0.991
	308K	Q_m_ = 399.42 mg·g^−1^	K_L_ = 0.0014 L·mg^−1^	0.989
	318K	Q_m_ = 423.37 mg·g^−1^	K_L_ = 0.0015 L·mg^−1^	0.997
Freundlich	298K	K_F_ = 1.131 mg·g^−1^	n = 0.789	0.988
	308K	K_F_ = 1.423 mg·g^−1^	n = 0.773	0.984
	318K	K_F_ = 1.637 mg·g^−1^	n = 0.768	0.998

**FIGURE 3:**
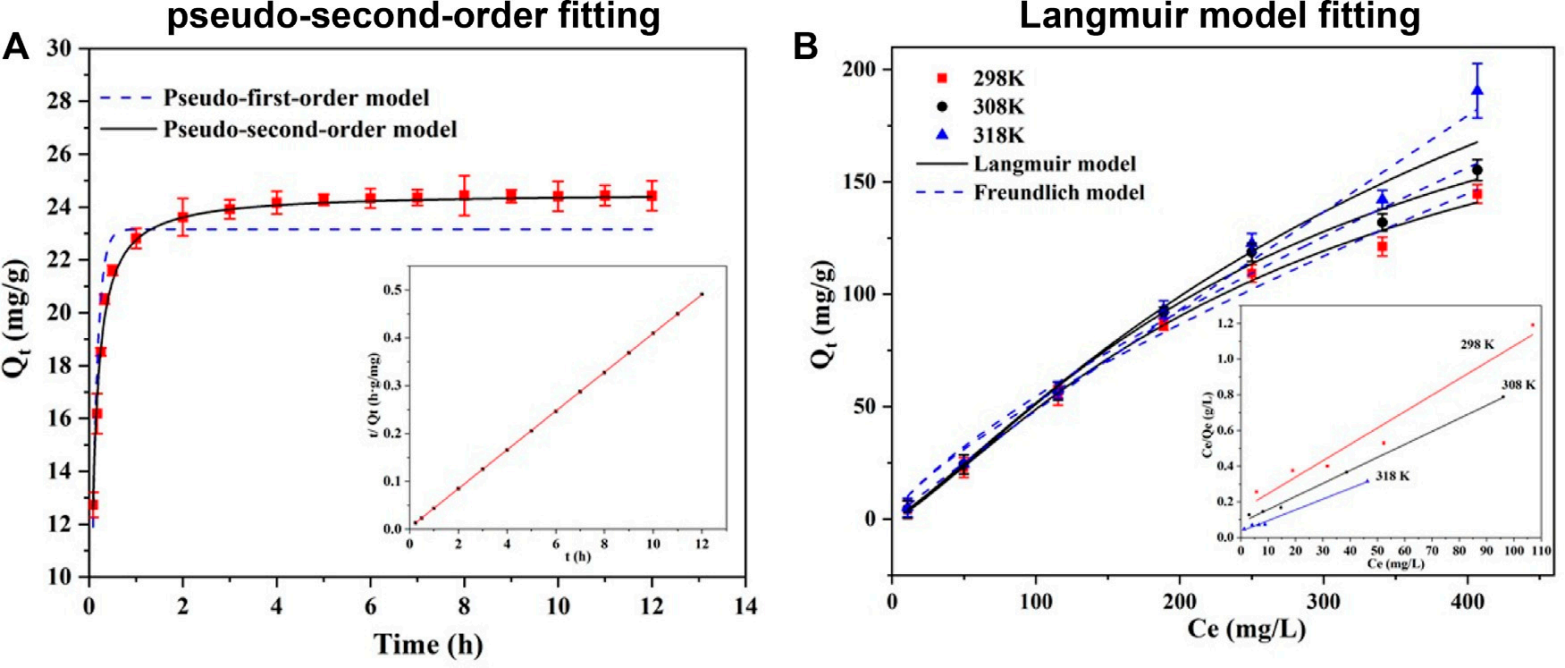
Fitting picture of kinetic **(A)** and isothermal models **(B)** for the adsorption of Se(IV) by CSBC.

The Langmuir and Freundlich models were applied to fit the adsorption isotherm data, as shown in Figure 3. The coefficient obtained by the Langmuir model (0.993) was higher than that of the Freundlich model (0.982). This demonstrates that this adsorption mainly occurred on the monolayer, with an estimated capacity of 190.53 mg/g. Furthermore, the value of R_L_ represented the affinity between Se(IV) and CSBC, while R_L_>1,R = 1, 1 > R > 0, and R = 0 were associated with the poor adsorption, linear adsorption, better adsorption performance, and irreversible adsorption, respectively (Ifthikar et al., 2021). Because these values are within the range of 0.768–0.789, the inter-surface reaction between CSBC and contaminants was favorable for adherence.

Thermodynamic analysis was employed to investigate whether the reaction was spontaneous and endothermic, as depicted in Figure 3 and calculated in Table 3. Based on the calculated results, the decreasing of Gibbs free energy (*ΔG*) within the increasing of reaction temperature from −7.13 to −10.53 kJ/mol suggests that the Se(IV) adsorption was spontaneous and the higher reaction is conductive for removal. The enthalpy change (Δ*H*) revealed the endothermic nature of adsorption, while the capacity could be accelerated with higher system temperature. The entropy change (ΔS) is positive. Hence, the Se(IV) elimination by CSBC was an endothermic and spontaneous process that involved many mechanisms, which needs further investigation.

**TABLE 3:** Thermodynamic analysis and fitting results.

*ΔH* (KJ·mol^−1^)	*ΔS* (KJ·mol^−1^)	*ΔG* (KJ·mol^−1^)		
		298 K	308 K	318 K
43.53	0.17	−7.13	−8.83	−10.53

### Removal mechanisms

The removal mechanisms of Se(IV) were elucidated by various techniques. First, the variations of the crystalline structure of adsorbents were investigated (see Supplementary Data). The generated 31.26°, 36.34°, and 52.18°peaks indicate the presence of Se^0^ according to the databases. These results indicate that chemical reduction occurred between Se(IV) and Fe^0^, which was also reported by other groups (Wei et al., 2021). The generation of Fe_2_O_3_ and Fe_3_O_4_ minerals could also support this speculation on the existence of Fe oxidation. More importantly, the obvious peaks with 2θ of 43.32° and 64.32° correspond to the Cu_2_Se crystalline structure. This phenomenon could be explained by the familiar chemical properties of sulfur and selenium (i.e., they belong to the elemental sixth-row main group), and the Se(IV) species were reduced with the oxidation of iron materials.

The variations of chemical states of surface elements were further investigated by XPS spectra. In the case of CSBC, the peaks with binding energies of 711.08, 713.48, 719.88, 724.28, and 727.68 eV were associated with the iron states of Fe^2+^, Fe^3+^, Fe^0^, Fe^2+^, and Fe^3+^, respectively. After adsorption, the molar ratios of Fe^0^ decreased from 9.81% to 6.91%, while the value of Fe2+ decreased from 60.85% to 55.24.31%, and that of Fe^3+^ increased from 29.31% to 37.85%. These results provide direct evidence for the chemical reactions between Se(IV) and CSBC. Moreover, the detailed XPS spectra of selenium after adsorption was also investigated (see Supplementary Data). In total, 48.04% Se(IV) pollutants were reduced into Se(0) and Se(-II) after adsorption, which was in accordance with the oxidation of Fe^0^ species. Furthermore, the C1s spectra were also depicted (see Supplementary Data). The peaks with the binding energies of 283.78, 285.26, 287.08, 288.98, and 292.88 eV, referred to as C=C, C-O, C=O, O-C=O, and π–π, respectively, while the variations of molar ratios of different carbon structure indicate the function of surface groups on Se(IV) species. Meanwhile, from the spectra of Cu 2p, the Cu(I) was observed and further proved the formation of Cu_2_Se co-precipitants, which is in accordance with XRD results.

The FT-IR results offer evidence for the reactions between Se(IV) and functional groups and also apply for the mechanism analysis (see Supplementary Data). As shown, the wavenumbers of 3314 and 1642 cm^−1^ were related to the stretching vibration of hydroxyl (-OH) and carbonyl (C=O) groups. Meanwhile, the C-H vibration in different groups as C=C, C-O, and C-H correspond with the wavenumbers of 1394, 1084, and 876 cm^−1^, respectively. These results suggest the abundant functional groups on the surface of CSBC. The peaks of Fe-O with wavenumber of 550 cm^−1^ also proved the successful loading of these functional species. After adsorption, obvious shifting and weakening were observed with the wavenumber of 3291, 1626, 1398, and 1115 cm^−1^, based on former analysis. These results prove the involvement of various oxygen-contained functional groups during Se(IV) elimination.

**FIGURE 4:**
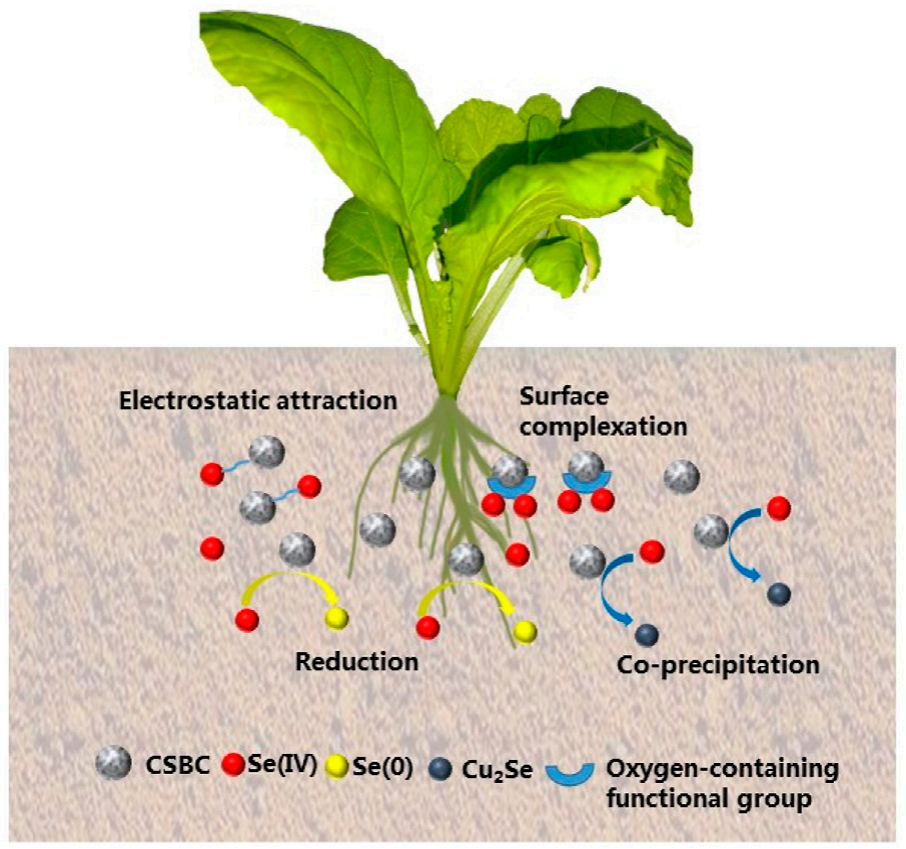
Mechanism of Se(IV) adsorption by CSBC.

Based on various characterizations after adsorption, the Se(IV) elimination process by CSBC can be attributed to the co-precipitation, chemical reduction, formation of complexations, electrostatic attraction, and so on. These mechanisms contribute to the acceptable capacity for Se(IV) immobilization. The mechanisms of adsorption on Se(IV) are shown in Figure 4. A comparison of removal capacity with other bio-adsorbents is given in Table 4. These results prove that these industrial product-derived functional materials could be promising adsorbents for selenite pollution control. Therefore, further evaluation should be conducted for its future application.

**TABLE 4:** Comparison of adsorbents for Se(IV) removal.

Adsorbents	Carbon substrate	Fabricants	Preparation method (main step)	Experimental conditions			Capacity (mg/g)	Ref.
				Initial pH	Initial concentration (mg/L)	Adsorbent dosage (g/L)		
Fe-BC	Purchased AC	Magnetite	Wet chemistry	5.3	0.2	0.2	1.26	Kwon et al. (2015)
Fe_2_O_3_-CNT	Carbon nanotubes	Iron (III) nitrate	Wet chemistry	6.0	0–40	0.2	111.00	Bakather et al. (2017)
Ca/P-BC	Date palm	Hydroxy apatite	Wet chemistry	5.0	50–200	2.0	57.27	Li et al. (2022)
UiO-66-BC	Purchased AC	MOFs	Dispersion and formation	7.0	0.1–200	1.0	168.00	Solis et al. (2020)
CS-PEI-GO	Graphene oxide	Chitosan and polymer	Surface modification	4.0	5–250	30.0	1.62	Bandara et al. (2019)
PAA-MGO	Graphene oxide	Fe salts and polymer	Co-precipitation	5.8	0–140	0.1	120.10	Lu et al. (2017)
LDHs-GO	Graphene oxide	MgAl-LDH	Wet chemistry	6.0	7.9–39.5	0.2	65.90	Koilraj et al. (2018)
PAMAM-GO	Graphene oxide	Polymer	Dispersion and formation	6.0	0–100	0.5	60.90	Xiao et al. (2016)
Fe-GO	Graphene oxide	Iron (III) nitrate	Wet chemistry	2.0	0.003–0.015	0.3	18.69	Koudelkova et al. (2019)
ZVI/BC	Brew waste	FeSO_4_ & NaBH_4_	Fe loading and reduction	4.0	10–100	2.8	34.77	Suganya and Senthil Kumar, (2019)
CSBC	Rice straw	Copper slag	Carbothermal reduction	2.0	100	2	190.53	This study
				4.0	100	2	30.17	

### Agro-industrial feasibility assessment

**FIGURE 5:**
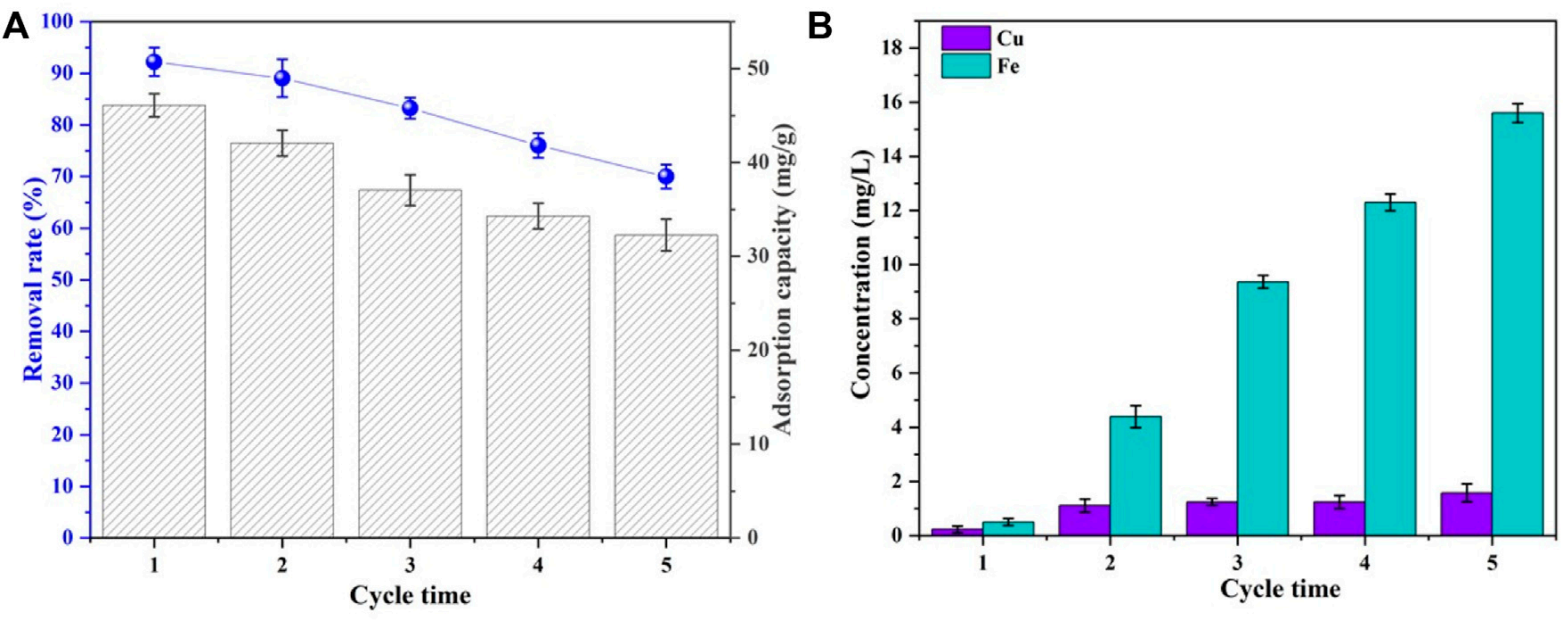
Removal rates and adsorption capacity **(A)** and leaching of Cu and Fe at each cycle of CSBC **(B)**.

The recyclability and leaching performance of the adsorbents are given in Figure 5. The Se(IV) removal rate and adsorbing capacity by CSBC were decreased from 92.2% to 70.01% and 46.07 to 32.27 mg/g after adsorption–desorption recycled for 5 times, respectively. This indicates the acceptable reusability of CSBC. Meanwhile, the leaching inherent metal ions were also detected as 0.23, 0.98, 1.11, 1.24, and 1.58 mg/L for Cu and 0.5, 4.39, 9.37, 12.31, and 15.62 mg/L for Fe. These values all met the national standard in China (GB 8978–1996) on wastewater effluent. One of the major concerns is the leaching of As from copper slag to the environment. However, in our study, the leaching of As was almost neglectable. This indicates the capsuling effect of biochar on the heavy metals, which is similar to other research (Li G et al., 2021). Simultaneously, the leaching concentrations of other heavy metals (Pb, Zn, Cd, and Cr) were measured, and the results are shown in Supplementary Table S1. The leaching concentrations of major heavy metals were within the range of the municipal wastewater quality discharge A standard (CJ343-2010). This indicates the reliability of CSBC as a functional adsorbent in practical applications.

The economic cost of CSBC is important for its further large-scale application. Due to the solid waste nature of CS and tobacco straw, the major CSBC preparation cost would be incurred by the pyrolysis processes. Hence, considering the labor costs and energy consumption, these values could vary from region to region. For example, the Taupo Carbon Producers prepared biochar at a cost of 144 USD per ton (Das et al., 2016), but this unmodified material exhibited limited performance when used for pollution control (Suliman et al., 2016). According to a field study, biochar prepared from 358 to 728 USD per ton could obviously enhance rice production and the ratio of bio-adsorbent to soil played a critical role (Wang H et al., 2018). Furthermore, Fe^0^-modified biochar exhibited better performance but normally higher preparation cost is involved, including chemicals such as iron salts and reductants (NaBH_4_ or hydrogen) (Kamali et al., 2019). Based on this analysis, the synthesis of CSBC was: 80 (Raw materials) +105 (Chemicals) +12 (Electricity) = 197 USD. This relatively acceptable cost was related to the facile steps and little labor force needed for CSBC, while chemical reductants were avoided and industrial by-products CS were reused as an iron source, which dramatically reduced the preparation cost (Kamali et al., 2022).

**FIGURE 6:**
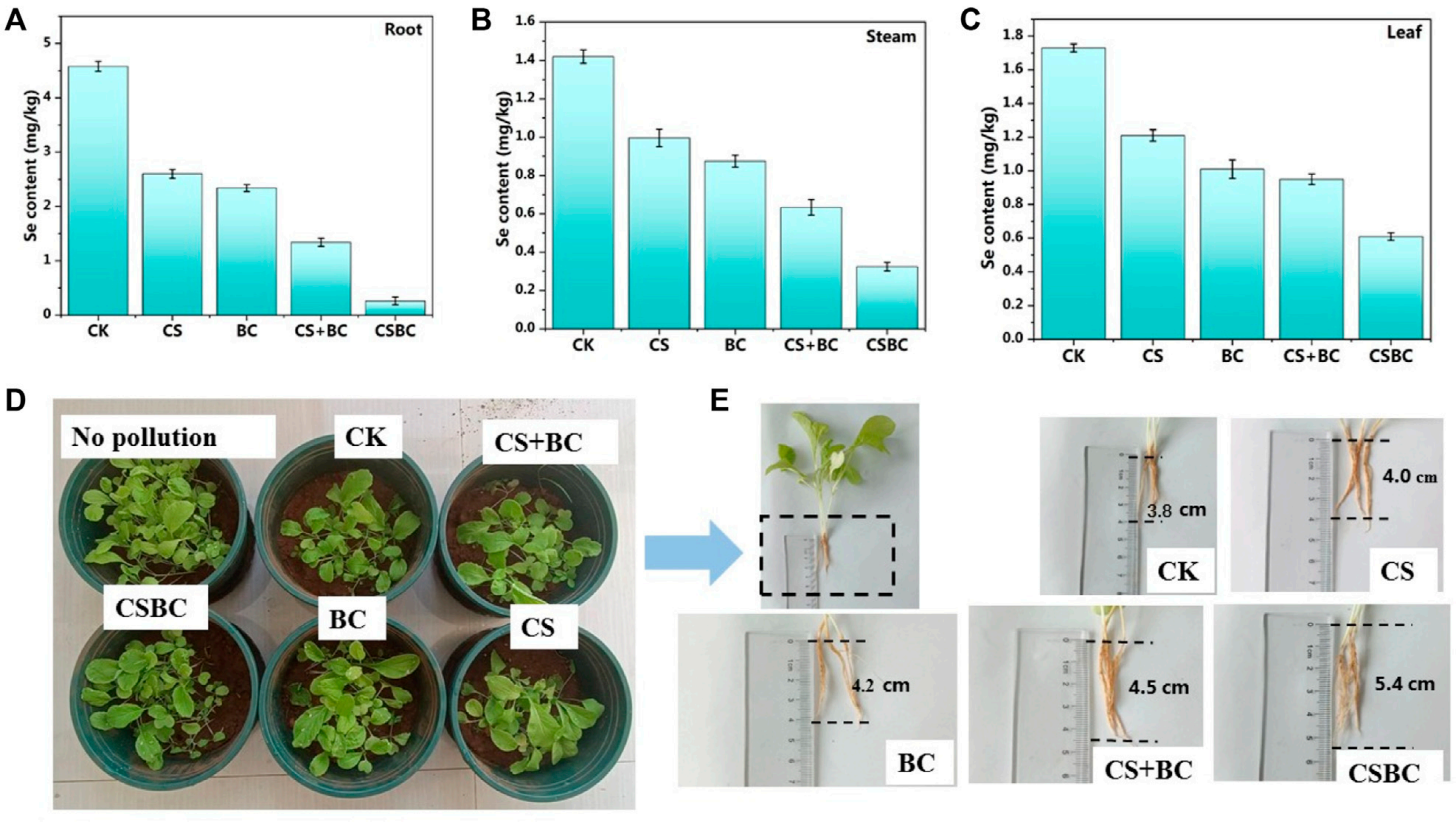
Se(IV) content in roots **(A)**, stems **(B),** and leaves **(C)** and growth picture **(D) (E)** of bok choy under various adsorbent treatments.

The soil remediation experiments of CSBC800-1 were conducted, and the results are given in Figure 6. Most of the bok choy plants were hypogenetic, and some were almost dead and sparse, which could be caused by the selenite contamination. However, for samples repaired by CSBC, the bok choy growth was more luxuriant. This could be explained by the mediation effects of CSBC functional materials. The heavy metal contents in the roots, stems, and leaves were also tested, and the results are given in Figure 6. The Se content in roots, stems, and leaves of bok choy treated by CSBC was the lowest and the root growth was the longest when compared with other restorative agents. This indicates that CSBC could reduce the bioavailability of Se (Łabaj et al., 2021). Based on these pot experiments, the CSBC exhibited potential soil remediation capability and inhibited bioaccumulation on bok choy to suppress heavy metal transfer between the biosphere and soil.

In summary, based on agro-industrial practicality evaluation, the CSBC exhibited promising and potential applicability with advantages of recyclability, cost-effectiveness, and plant-growth enhancement. Therefore, the direct and indirect benefits of CSBC for agricultural and wastewater purification highlight its potential future application.

## Conclusion

In this work, tobacco straw and copper slag were employed for preparation of iron-fabricated biochar to eliminate Se(IV) from wastewater. The physicochemical characterizations indicate the successful loading of iron species. The Se(IV) removal process was fitted by the kinetics and isotherm adsorption model. The removal was endothermic and spontaneously occurred on the monolayer dominated by chemisorption, with the mechanisms of electrostatic attraction, precipitation, chemical reduction, and formation of complexations. The existence of Cu_2_Se indicates the function of copper ions in co-precipitation with reduced Se ions. These results prove the feasibility of this copper slag and tobacco straw-derived bio-adsorbent in selenium-contained wastewater purification.

## Data Availability

The original contributions presented in the study are included in the article/Supplementary Material; further inquiries can be directed to the corresponding author.
