# Supplementary material for: Selenite elimination via zero-valent iron modified biochar synthesized from tobacco straw and copper slag: Mechanisms and agro-industrial practicality
Source: Front Bioeng Biotechnol. 2022 Nov 14;10:1054801. doi: 10.3389/fbioe.2022.1054801 (PMC9701720; doi:10.3389/fbioe.2022.1054801)
Supplement: Supplementary file 1 [file DataSheet1.docx]

**Selenite elimination via zero-valent iron modified biochar synthesized from tobacco straw and copper slag: Mechanisms and agro-industrial practicality**

**Qiong Luo^1^, Dingxiang Chen^1^, Ting Cui, Ran Duan, Yi Wen, Fang Deng, Lifang Li, Huabin Wang*, Yong Zhang*, Rui Xu***

School of Energy and Environment Science, Yunnan Normal University, Kunming 650500, P. R. China;

Yunnan Key Laboratory of Rural Energy Engineering, Kunming 650500, P. R. China;

* Corresponding author: Tel: +86 871 65940928; Fax: +86 871 65940928

1. mail address: ecowatch_xr@163.com (Rui Xu)

Qiong Luo1 and Dingxiang Chen1 These authors contributed equally to this work and share first authorship

# Figures and tables captions

**Table S1.** Heavy metal concentrations of CSBC after leaching tests.

**Fig. S1.** SEM image of the BC (a), CS (b), CSBC (c) and EDS (d), mapping image of CSBC.

**Fig. S2.** N_2_ adsorption-desorption isotherm picture of CSBC.

**Fig. S3.** XRD patterns of CSBC at different temperatures (a) and before and after adsorption (b).

**Fig. S4.** XPS spectra of C, Cu, Fe before (a) and after (b) CSBC adsorption, XPS spectra of full spectrum before (c), after (d),by CSBC adsorption and XPS spectrum of Se (e)

**Fig. S5.** FTIR spectra of CSBC before and after adsorption.

**Table S1.** Heavy metal concentrations of CSBC after leaching tests (mg/L).

|  | Cr | Cu | Pb | Zn |
| --- | --- | --- | --- | --- |
| Content | 0.03 | 0.09 | 0.17 | 0.49 |


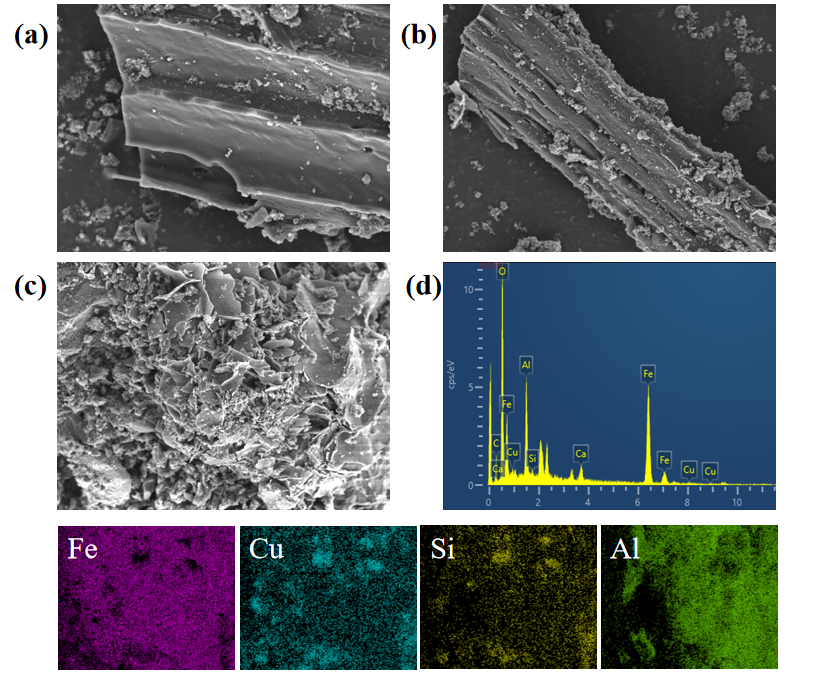
**Fig. S1** SEM image of the BC (a), CS (b), CSBC (c) and EDS (d), mapping image of CSBC.

**
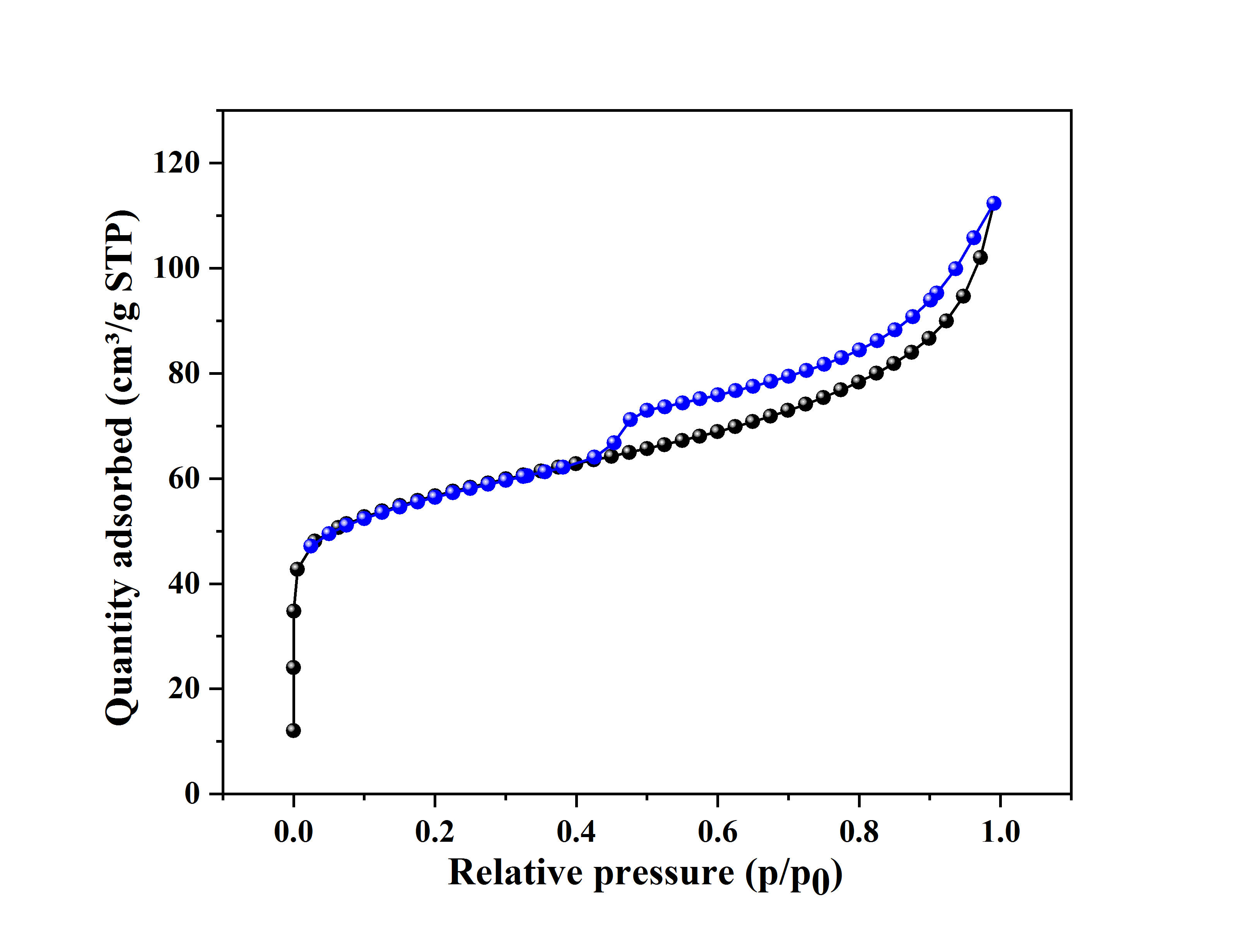
Fig. S2** N_2_ adsorption-desorption isotherm picture of CSBC.

**
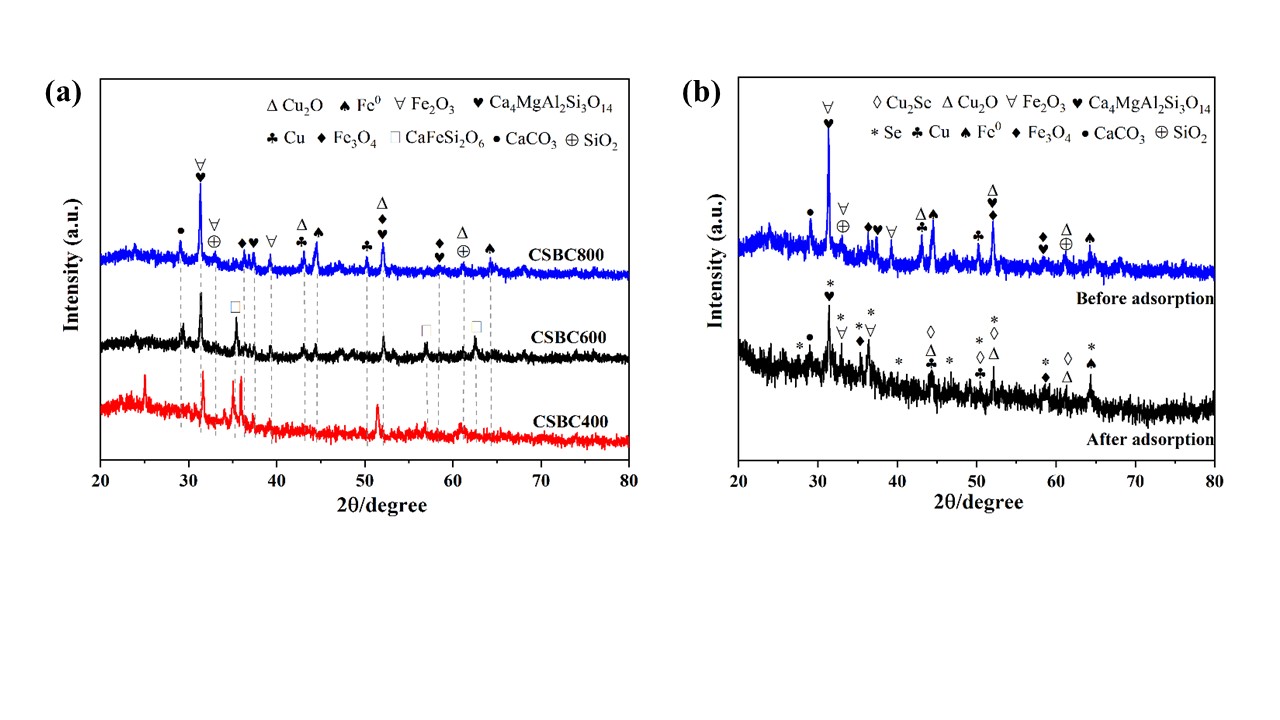
Fig. S3** XRD patterns of CSBC at different temperatures (a) and before and after adsorption (b).

**
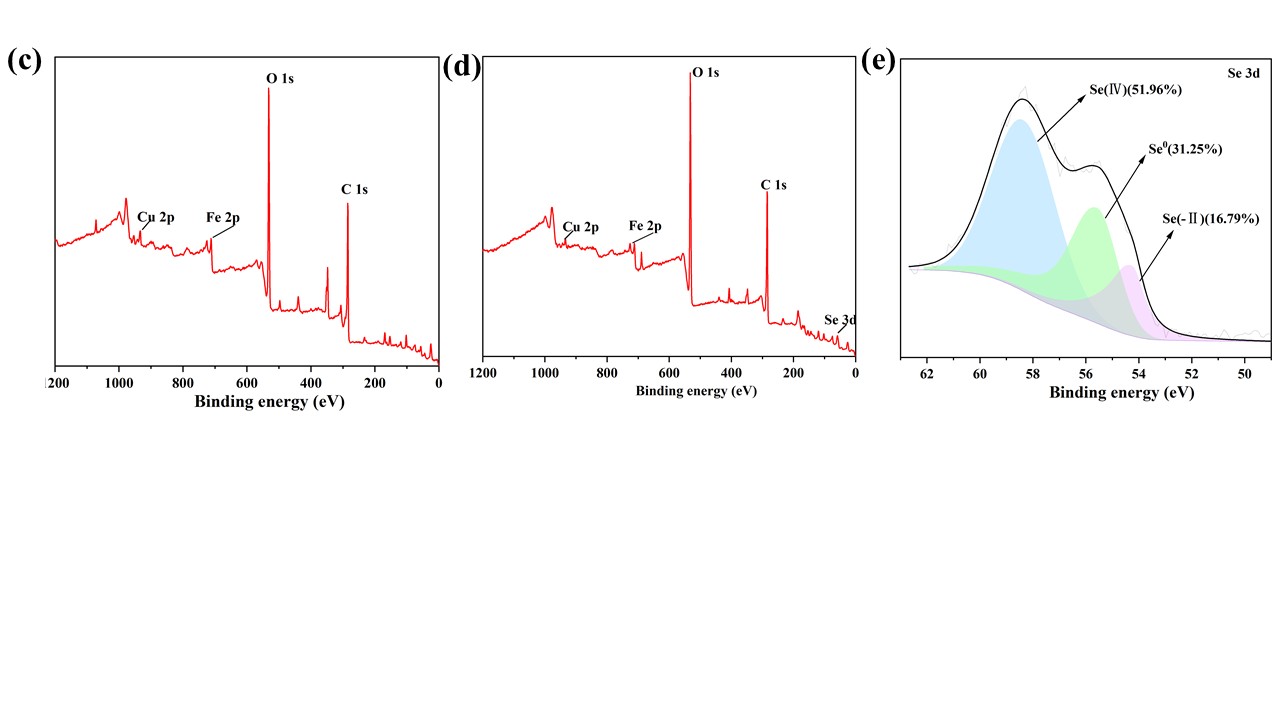

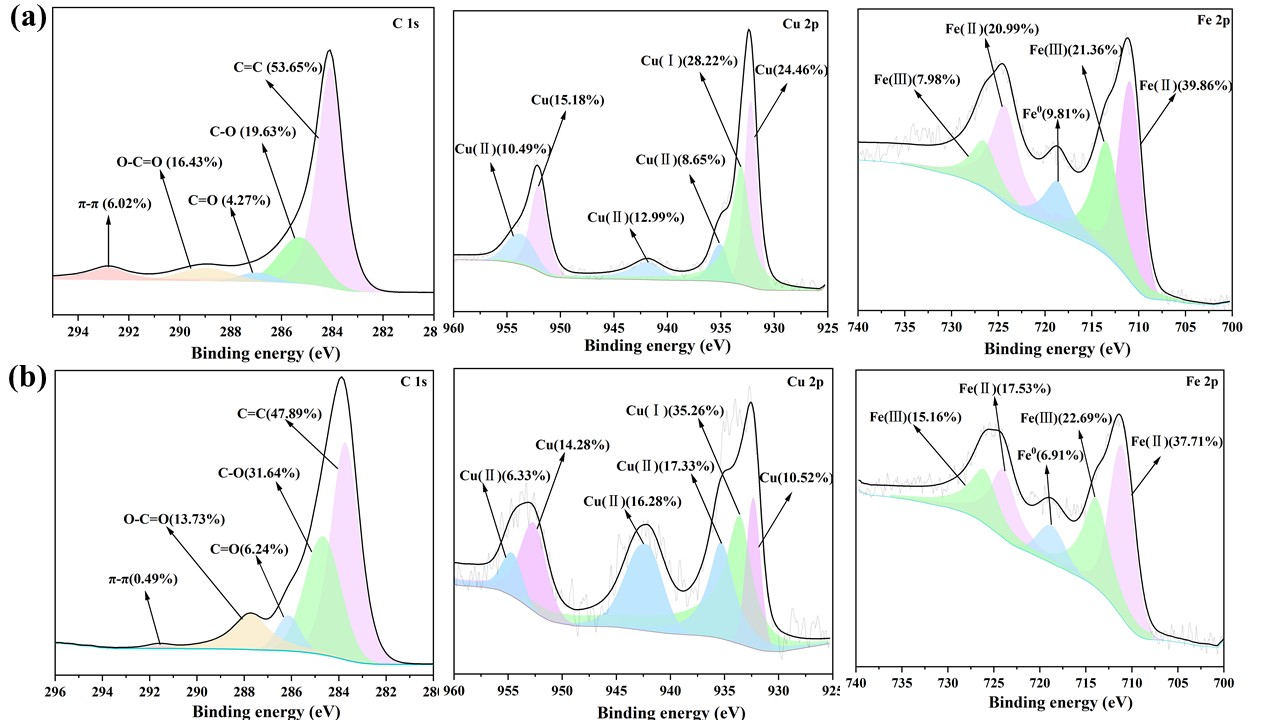
Fig. S4** XPS spectra of C, Cu, Fe before (a) and after (b) CSBC adsorption, XPS spectra of full spectrum before (c), after (d),by CSBC adsorption and XPS spectrum of Se (e).

**
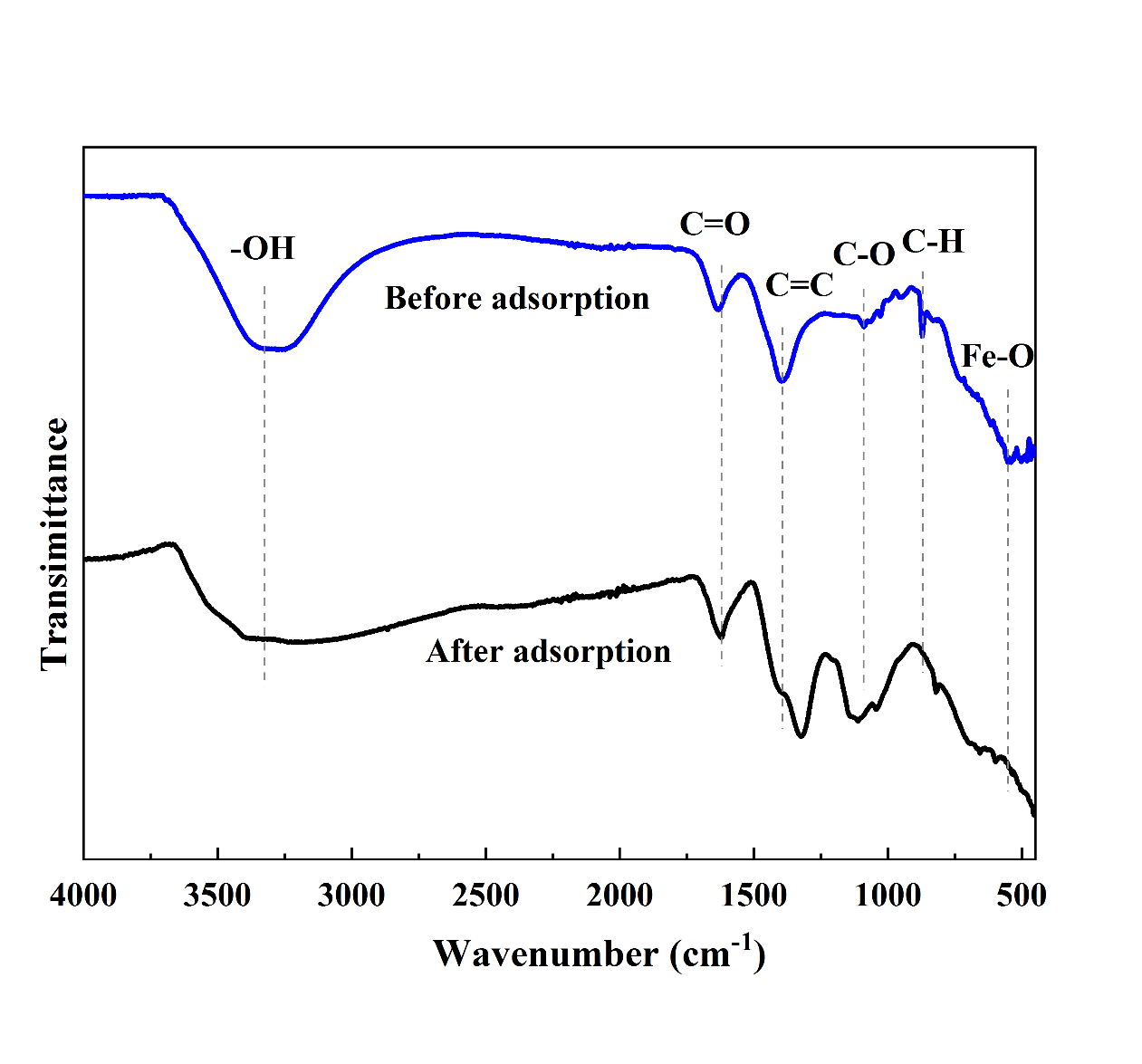
Fig. S5** FTIR spectra of CSBC before and after adsorption.
